# Polymeric immunoglobulin receptor (PIGR) exerts oncogenic functions via activating ribosome pathway in hepatocellular carcinoma

**DOI:** 10.7150/ijms.49790

**Published:** 2021-01-01

**Authors:** Yuan Zhang, Jijie Zhang, Xiaorong Chen, Zongguo Yang

**Affiliations:** 1 Department of Integrative Medicine, Shanghai Public Health Clinical Center, Fudan University, Shanghai 201508, China.; 2 Department of Oncology, The People's Hospital of Danyang, Affiliated Danyang Hospital of Nantong University, Jiangsu 212300, China.

**Keywords:** polymeric immunoglobulin receptor, hepatocellular carcinoma, disease-free survival, recurrence, ribosome

## Abstract

**Objective:** This report aimed to investigate the potential mechanism of polymeric immunoglobulin receptor (PIGR) in promoting cancer development in hepatocellular carcinoma (HCC).

**Methods:** PIGR expression was investigated in Gene Expression Omnibus (GEO), Oncomine, The Cancer Genome Atlas (TCGA) and The Human Protein Atlas (HPA) databases. Relationships between PIGR and HCC survival and clinico-pathological features were conducted in TCGA. RNAseq of PIGR overexpression and knockdown samples in Bel-7404 cells were performed for identifying potential mechanisms.

**Results:** PIGR was significantly overexpressed in tumors compared to nontumors and in HCC serum peripheral blood mononuclear cells (PBMC) than in healthy individuals (all *p* < 0.05). In TCGA, PIGR was highly altered in 14% HCC patients. PIGR upregulation was significantly associated with poor disease-free survival (*p* < 0.05). More patients recurred/progressed in PIGR altered group compared to unaltered group (*p* < 0.01). PIGR was significantly higher in HCC patients with incomplete cirrhosis (*p* < 0.001) and established cirrhosis (*p* < 0.05). Fewer patients had N_0_ lymph node stage in PIGR altered group than those in the unaltered group (*p* < 0.05). PIGR RNAseq revealed that ribosome signaling was the common pathway in PIGR overexpression and PIGR knockdown samples. RNAseq analysis indicated that RPL10, RPL10A, RPL12, RPL19, RPL36, RPL38, RPL41, RPL6, RPL8, RPS12, RPS14, RPS15A, RPS2, RPS27A and RPSA were significantly upregulated in PIGR overexpression group and downregulated in PIGR underexpression group (all *p* < 0.05).

**Conclusions:** Aberrant PIGR was associated with HCC recurrence, and PIGR stimulated ribosome pathway might be a potential mechanism.

## Introduction

Liver cancer, comprising 75%~85% cases of hepatocellular carcinoma (HCC), is predicted to be the sixth common and the fourth lethal cancer worldwide 1-3. During the past two decades, there has been a marked increase in HCC incidence and cancer related annual deaths 3, 4. A series of complex multistep hepatocarcinogenesis mechanisms of angiogenesis, invasion, metastasis, regulation of cell cycle, proliferation, differentiation, cell invasion, and inflammation were involved in HCC progression 5, 6. However, the details and mechanisms of hepatocarcinogenesis still remain to be elucidated.

Solely produced by intestinal epithelial cells, polymeric immunoglobulin receptor (PIGR) captured and transcytosed dimeric IgA (dIgA) from lamina propria to intestinal lumen across epithelial cells, participating in mucosal immune system 7, 8. Previous evidence indicated that proinflammation cytokines could activate Janus kinase-signal transduction and activator of transcription (JAK-STAT), NF-κB and mitogen-activated protein kinase (MAPK) signaling pathways, leading to PIGR overexpression 7, 8. Moreover, downregulation of PIGR resulted in inhibition of cell proliferation, adhesion and migration in pancreatic ductal adenocarcinoma (PDAC) 9. Recently, PIGR has been proved to be involved in the human tumorigenesis and malignancies 9-12. As a vital inflammatory mediator 7, PIGR played an important role in hepatitis B virus (HBV) infection, chronic liver inflammation, tumor growth, recurrence, and metastatic progression in HCC 10-12. Mechanistically, Smad induced epithelial-mesenchymal transition (EMT) 10 and Rac1/CDC42-MEK/ERK cascade 12 were proved to be potential mechanisms of PIGR related cancer malignancy in HCC.

As part of intestinal immune network for IgA production pathway, we previously found that PIGR was overexpressed in HCC tumors 13. In this analysis, we aimed to investigate the oncogenic functions of PIGR and the potential mechanism of PIGR in promoting malignancy in HCC.

## Materials and Methods

### Ethical Statement

Our study did not require an ethical board approval because it did not contain human or animal trials.

### Data sources

Studies compared PIGR between cancer and normal samples in liver cancer were selected with analysis type by “liver cancer vs. normal”, gene name by “PIGR”, threshold by *p*-value ≤ 1E-4, fold change ≥ 2 and top 10% gene rank in Oncomine database (https://www.oncomine.org/). Profiles including Guichard Liver, Guichard Liver 2 and TCGA liver were included in this analysis. In order to validate the PIGR expression levels in serum and tissues HCC and healthy candidates, Gene Expression Omnibus (GEO, https://www.ncbi.nlm.nih.gov/geo/) database was searched using heading terms including “liver cancer” and “hepatocellular carcinoma”. All the series with expression profiling by array were included and homo sapiens were selected. Platforms with terms including “ID”, “Gene Symbol” and “ENTREZ_GENE_ID” were also restricted. GEO profiles including GSE49515 14, GSE55092 15 and GSE60502 16 were included in this analysis. Additionally, PIGR protein detected by immunohistochemistry was obtained from the Human Protein Atlas (HPA) databases (http://www.proteinatlas.org) 17-19.

### Bioinformatics analysis

Raw.CEL files of the microarray from each GEO dataset were normalized by quantile method of Robust Multichip Analysis (RMA) from R affy package 20. Gene expression of PIGR between tumor and nontumor tissues was performed by R Limma package 21. Series datasets with expression profiling by array was searched by gene name “PIGR” in GEO database, and GSE34630 22 profile was used for gene set enrichment analysis (GSEA) by GSEA software 23.

### Cell culture

Bel-7404 human HCC cell line was purchased from Chinese Academy of Sciences. Cells were cultured in Dulbecco's modified Eagles' medium (DMEM/high glucose) supplemented with 10% fetal bovine serum (FBS) (Cellsera, NSW, Australia) containing 50 µg/ml penicillin and 50 µg/ml streptomycin at 37°C in a 5% CO2 incubator. The medium was changed three times every week.

### Vector construction and transduction

A lentiviral PIGR short hairpin RNA (shRNA) was purchased from Genechem (Shanghai, China). The shRNA sequence targeting human PIGR complementary DNA were as follows:

- shRNA1-PIGR: TCGATCACTCAGGAGACAT;
- shRNA2-PIGR: CGTCTATGTGGCAGTTGAA;
- shRNA3-PIGR: CAACTATACAGGAAGAATA.

A scrambled shRNA was included as a negative control (NC). The PIGR overexpression plasmid was generated from human total cDNA. This construct was generated with the forward and reverse primers. Forward: 5' GAGGATCCCCGGGTACCGGTCGCCACCATGGCGGAGCCGAGCGGC3', reverse: 5' TCACCATGGTGGCGACCGGGCTGACACTCAACTGAGCA3'. The target sequence was inserted into GV248 lentiviral vector (Genechem, Shanghai).

### Transcriptome sequencing analysis

To identify mRNAs in Bel-7404 cells in response to PIGR challenges, mRNA libraries derived from three groups including PIGR overexpression group, PIGR knockdown group and negative control groups were constructed and sequenced using the Illumina Novaseq6000-sequencing platform. The mRNA sequencing analysis was conducted by Novogene (Tianjin, China).

### Quantitative real-time PCR (qRT-PCR)

Expression levels of genes screened by transcriptome sequencing analysis were verified by qRT-PCR according to the manufacturer's instructions of Takara (Takara Bio Inc, Shiga, Japan). The primer sequence of these genes for qRT-PCR was summarized in Table 1.

### Western blotting analysis

Western blot analysis was performed according to standard procedures using antibodies against PIGR (ImmunoWay Biotechnology, TX, USA) and actin (Cell Signaling Technology, MA, USA). The protein expression detected by western blot were analyzed and quantified using Image J software (https://imagej.nih.gov/ij/index.html).

### Survival analysis

Liver Hepatocellular Carcinoma (TCGA, Firehose Legacy) database in cBioPortal for cancer genomics web service was obtained 24, 25. PIGR mRNA expression levels calculated by log2 calculation were compared based on clinical attribute in HCC patients. To evaluate associations between PIGR and survival and clinic-pathological features in HCC patients, gene data with Z scores and clinical data of HCC patients in Liver Hepatocellular Carcinoma (TCGA, Firehose Legacy) database were downloaded from cBioPortal and matched using VLOOKUP index in EXCEL. According to the pathological diagnosis, 361 HCC patients were included in the analysis.

### Statistical analysis

Differences of variables between the individual groups were analyzed using student *t* test, Mann-Whitney *U*-test and Chi-square test based on variables types. The Kaplan-Meier method with log rank test was used to compare survival between different groups. Stata software version 16.0 (Stata Corp LLC, Texas, USA) was used. A two-tailed *p* < 0.05 were considered significant for all tests.

## Results

### PIGR expression levels between tumors and nontumors

**Figure 1:**
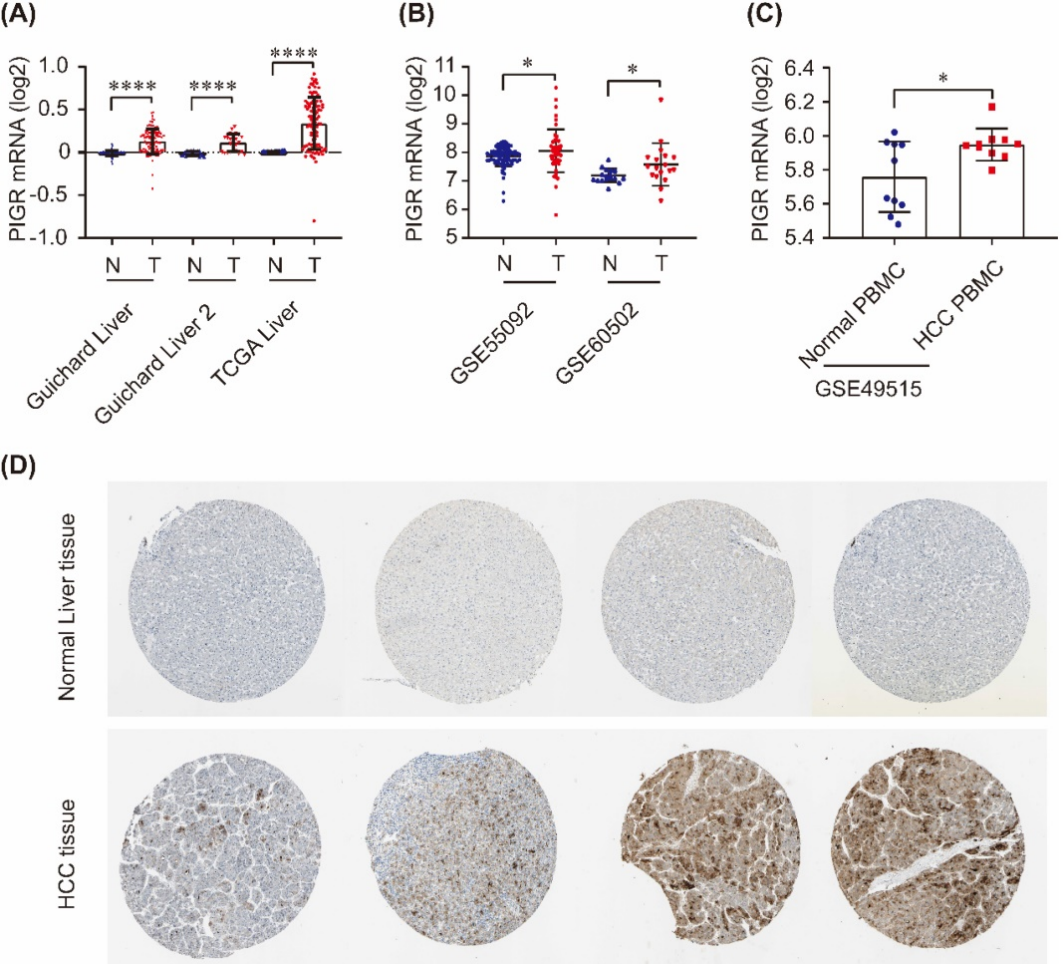
**PIGR expression in HCC tissues and serum samples.** PIGR mRNA was significantly upregulated in Guichard Liver, Guichard Liver 2 and TCGA Liver in Oncomine database (A), and in GSE55092 and GSE60502 in GEO database (B); PIGR mRNA was significantly overexpressed in HCC PBMC samples compared to that in healthy individuals (C); PIGR protein was highly expressed in HCC tumor tissues compared to that in normal liver tissues (D).* *p* < 0.05, ** *p* < 0.01, *** *p* < 0.001, **** *p* < 0.0001.

As shown in Figure 1, PIGR mRNA was significantly upregulated in tumor tissues compared to nontumor tissues in Guichard Liver, Guichard Liver 2 and TCGA Liver from Oncomine database (all *p* < 0.0001, Figure 1A), as well as in GSE55092 and GSE60502 profiles in GEO databases (both *p* < 0.05, Figure 1B). In serum samples, PIGR mRNA was significantly overexpressed in PBMC of HCC patients compared to that in healthy individuals (*p* < 0.05, Figure 1C). We also detected the PIGR protein levels by immunohistochemistry in HPA database. As shown in Figure 1D, PIGR protein was higher in tumor tissues than nontumor tissues in HCC patients, in consistent with our previous report 13.

### Associations between PIGR and survival and clinico-pathological features

**Figure 2:**
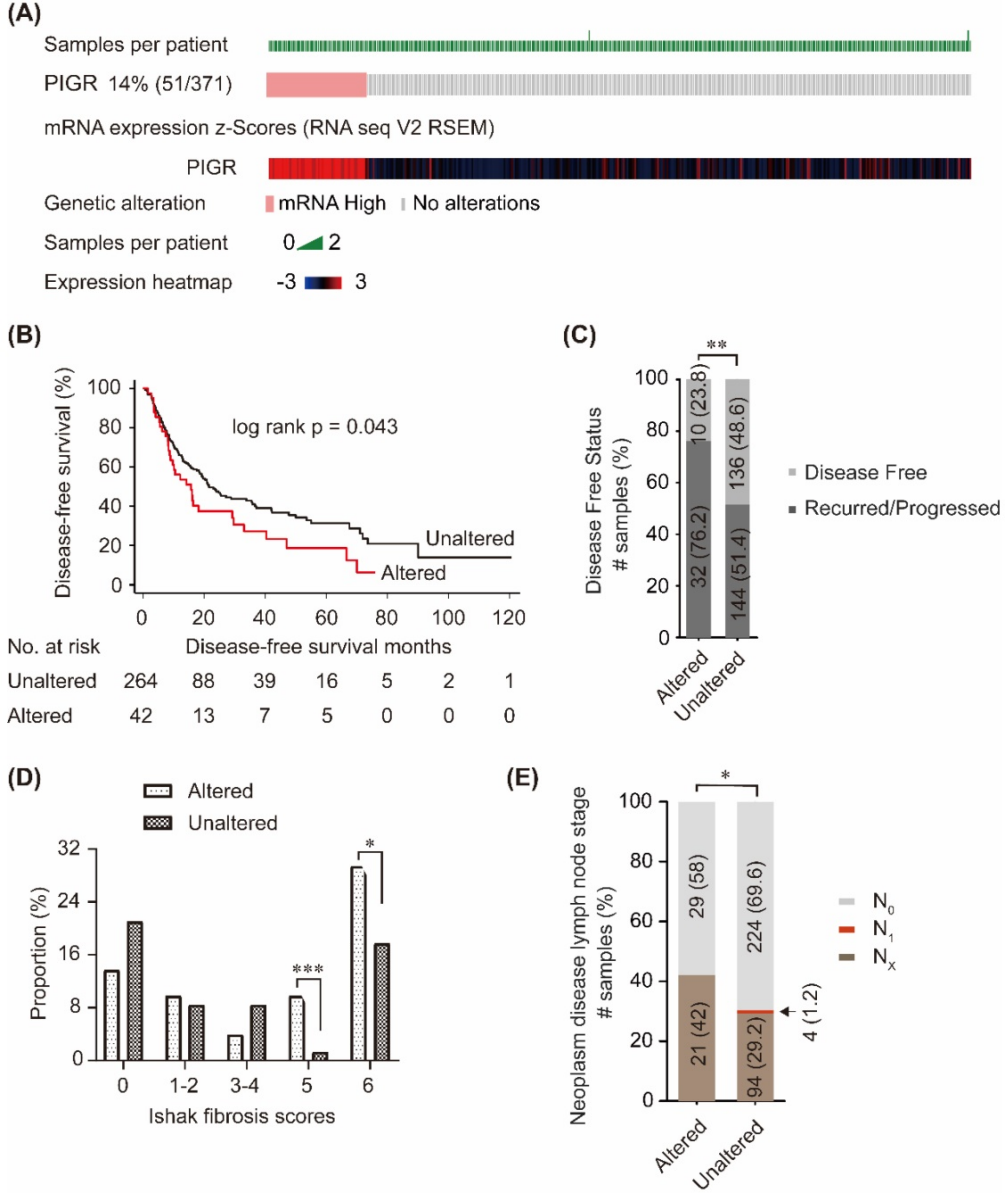
**Associations between PIGR and survival and clinico-pathological features in HCC patients from cBioPortal database.** 14% (51/371) HCC patients had PIGR highly altered in tumor tissues (A); High PIGR mRNA was significantly associated with worse disease-free survival in HCC patients (B); More patients developed recurrence/progression in PIGR altered group than those in unaltered group (C); PIGR was significantly higher in HCC patients with nodular formation and incomplete cirrhosis and established cirrhosis (D); Less patients had N_0_ lymph node stage in PIGR altered group than those in unaltered group by American Joint Committee on Cancer code (E). * *p* < 0.05, ** *p* < 0.01, *** *p* < 0.001, **** *p* < 0.0001.

In cBioPortal database, PIGR mRNA was upregulated in 14% (51/371) patients (Figure 2A). Patients without PIGR expression data were excluded, and 306 patients were included in the survival analysis. As shown in Figure 2B, high PIGR was significantly associated with worse disease-free survival (DFS) in HCC patients (*p* = 0.043, Figure 2B). In PIGR altered group, more patients developed recurrence/progression than those in unaltered group (76.2% vs. 51.4%, *p* < 0.01, Figure 2C). PIGR was significantly higher in HCC patients with nodular formation and incomplete cirrhosis (*p* < 0.001, Figure 2D) and established cirrhosis (*p* < 0.05, Figure 2D). Fewer patients had N_0_ lymph node stage in PIGR altered group than those in unaltered group by American Joint Committee on Cancer code (*p* < 0.05, Figure 2E).

### PIGR activated ribosome pathway

**Figure 3:**
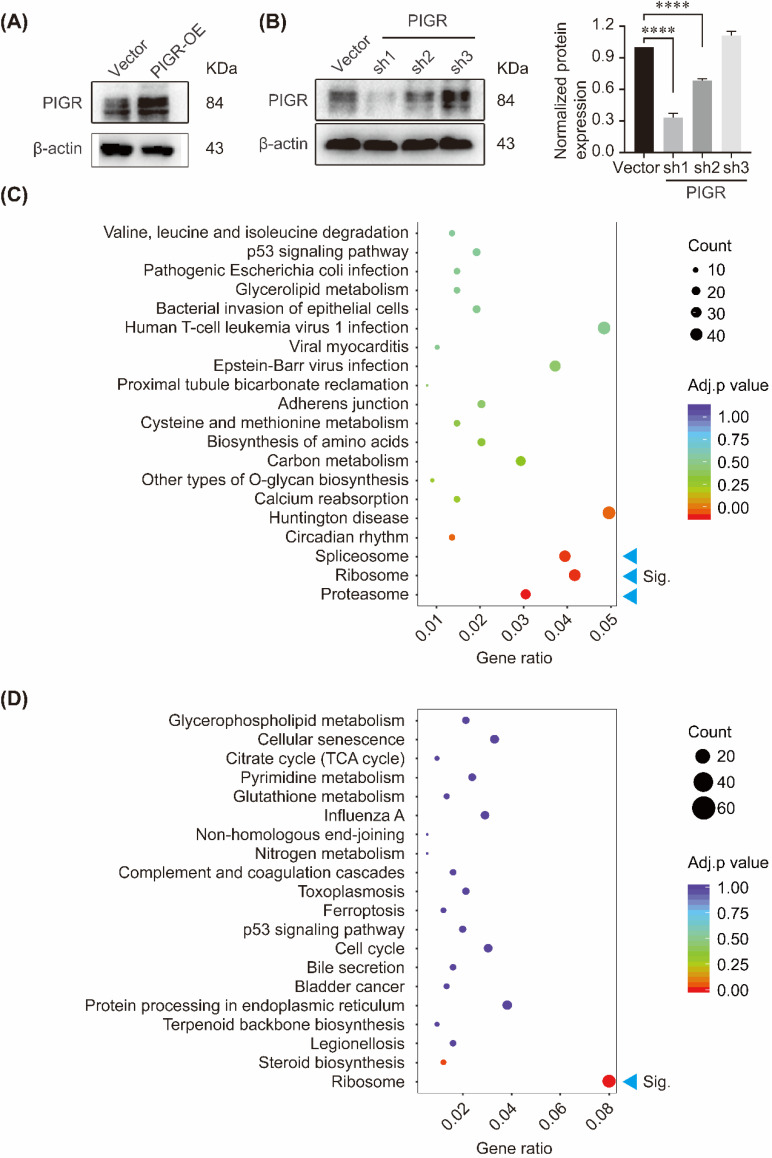
** KEGG enrichment of genes changed with PIGR expression.** The overexpression efficiency and knockdown efficiency of PIGR (A); Three pathway including spliceosome, ribosome and proteasome were significantly enriched in PIGR overexpression group (B); Ribosome pathway was significantly enriched in PIGR knockdown group (C). * *p* < 0.05, ** *p* < 0.01, *** *p* < 0.001, **** *p* < 0.0001.

**Figure 4:**
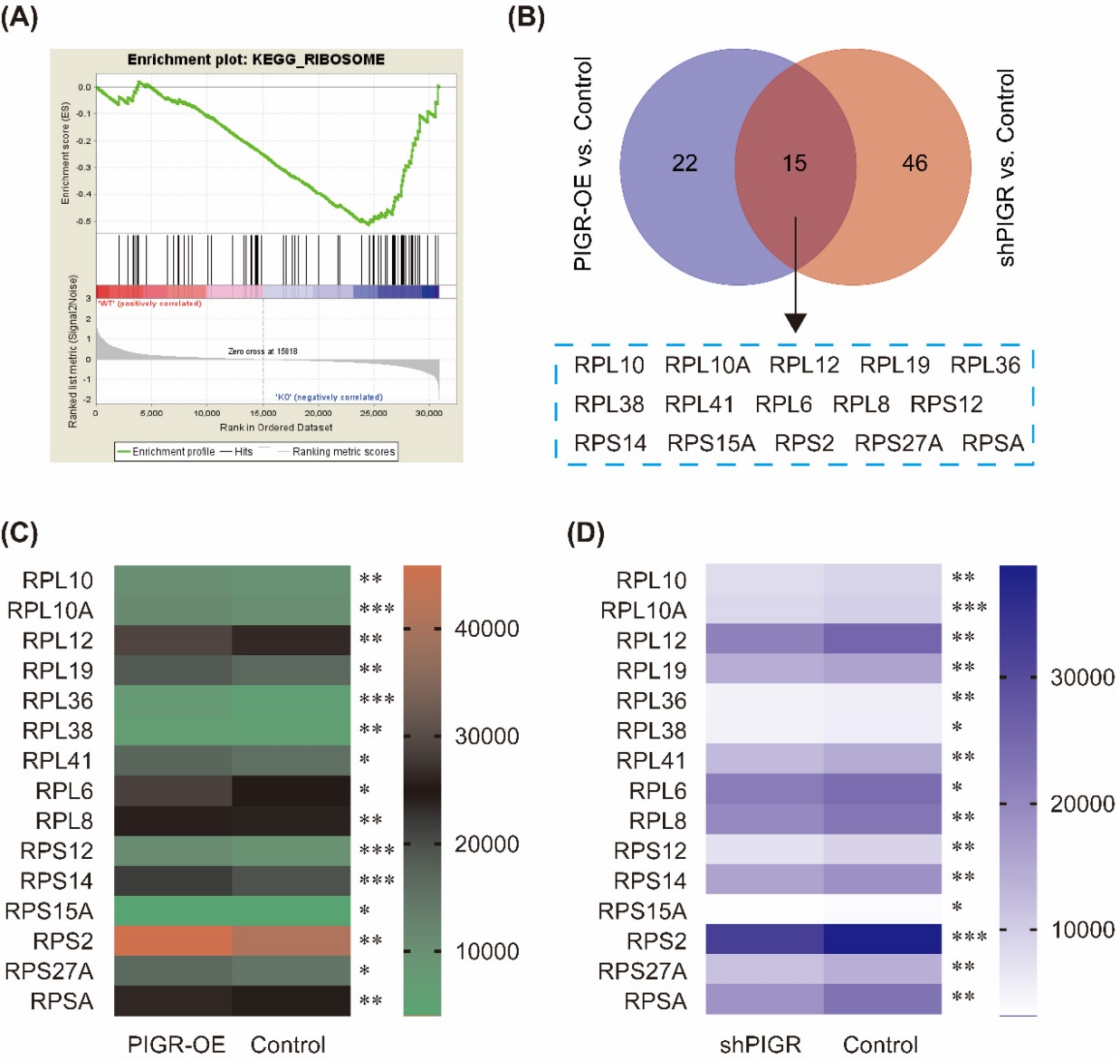
** Common genes changed with PIGR expression.** In GSE34630, wild type mice left untreated (5 replicates) and PIGR knockout mice left untreated (6 replicates) were included for GSEA enrichment. Ribosome pathway was one potential pathway induced by PIGR (A). According to the RNAseq results, 15 genes were common genes both in PIGR overexpression and knockdown groups compared to negative control group (B). All these 15 genes were significantly overexpressed in PIGR overexpression group (C), and downregulated in PIGR knockdown group (D). * *p* < 0.05, ** *p* < 0.01, *** *p* < 0.001, **** *p* < 0.0001.

We performed KEGG pathway enrichment in PIGR overexpression group and PIGR knockdown group using transcriptome sequencing analysis. The overexpression efficiency and knockdown efficiency of PIGR were shown in Figure 3A and 3B. The most effective short hairpin RNA (shRNA1-PIGR) of PIGR knockdown was selected for the transcriptome sequencing analysis (Figure 3B). Three pathway including spliceosome, ribosome and proteasome were significantly enriched in PIGR overexpression group (Figure 3C), while only ribosome pathway was significantly enriched in PIGR knockdown group (Figure 3D). In GSE34630, wild type mice left untreated (5 replicates) and PIGR knockout mice left untreated (6 replicates) were included for GSEA enrichment. As shown in Figure 4, ribosome pathway was one potential pathway induced by PIGR (Figure 4A). According to our RNAseq results, 15 genes including RPL10, RPL10A, RPL12, RPL19, RPL36, RPL38, RPL41, RPL6, RPL8, RPS12, RPS14, RPS15A, RPS2, RPS27A and RPSA were common genes both in PIGR overexpression and knockdown groups compared to negative control group (Figure 4B). All these 15 genes were significantly overexpressed in PIGR overexpression group (all *p* < 0.05, Figure 4C), and downregulated in PIGR knockdown group (all *p* < 0.05, Figure 4D).

**Figure 5:**
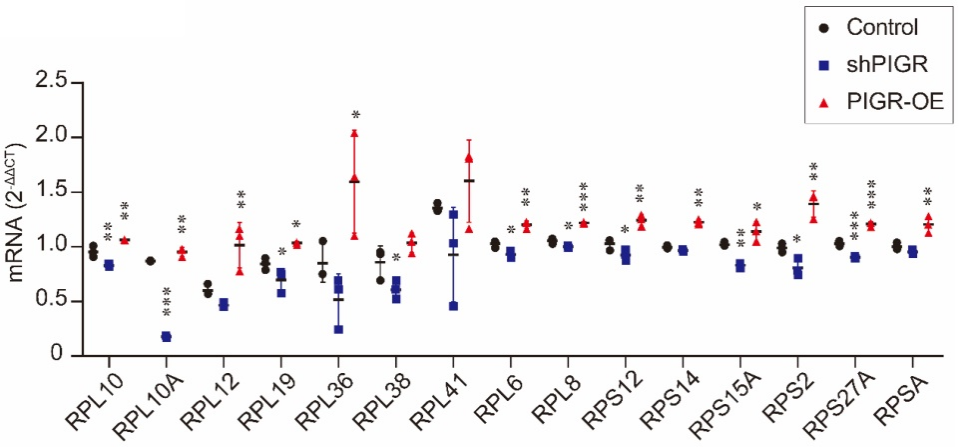
Expression levels of screened genes by RNAseq using qRT-PCR analysis. * *p* < 0.05, ** *p* < 0.01, *** *p* < 0.001, **** *p* < 0.0001.

In addition, we conducted qRT-PCR analysis to verify expression levels of these 15 genes. As summarized in Figure 5, RPL10, RPL10A, RPL12, RPL19, RPL36, RPL6, RPL8, RPS12, RPS14, RPS15A, RPS2, RPS27A and RPSA were all upregulated in PIGR overexpression group compared to those in negative control group (all *p* < 0.05, Figure 5). While RPL10, RPL10A, RPL19, RPL38, RPL6, RPL8, RPS12, RPS15A, RPS2 and RPS27A were all downregulated in PIGR knockdown group than those in negative control group (all *p* < 0.05, Figure 5).

## Discussion

In line with previous reports 10, 13, we found that PIGR was upregulated in tumor tissues and serum samples in HCC patients. In addition, high PIGR contributed to advanced liver fibrosis stage, low incidence of lymph node stage N_0_, and high risk of recurrence. Considered previous literatures and our results, we assumed that PIGR exerts as an oncogene in the development of HCC.

The extracellular portion of PIGR binding to dIgA is called the secretory component (SC), which is responsible for intracellular neutralization of some viruses 26, 27. Cytokines released by host and adaptive immune cells including interferon (IFN)-γ, interleukin (IL)-1, IL-4, IL-17, tumor necrosis factor (TNF) and lymphotoxin (LT)-β could also indirectly simulating the PIGR expression 28-30. Consequently, these cytokines involved in multiple intracellular signaling pathway including JAK-STAT, NF-κB and MAPK, resulting in upregulation of PIGR 7, 8. In PDAC, downregulation of PIGR results in reduction of cellular proliferation, adhesion and migration *in vitro* 9. *In vivo*, PIGR downregulation resulted in reduced cancer cell invasion and diminished stromal activity. And, low PIGR correlated with better survival in PDAC patients 9. Additionally, chronic inflammation induced by HBV might upregulate the expression of PIGR. PIGR upregulation increased the nuclear translocation of Smad2/3, leading to the activation of Smad pathway. Consequently, the transcriptional regulation mediated by Snail, Slug and ZEB1 leads to promotion of EMT 10, 11. All these underlying mechanisms supported the hypothesis that PIGR exerts an oncogenic function in the hepatocarcinogenesis.

Even though, the downstream signaling of PIGR remains to be elucidated. According to our results, we believed that PIGR might simulate ribosome pathway activation and promoted cancer progression. Ribosomes are required for protein production, and are involved in the process of cell proliferation, growth and survival 31. Current evidences have elucidated that inhibition of ribosome biogenesis could induce p53 stabilization and activation, mainly through ribosomal proteins (RPs)-MDM2-p53 pathway 32-34. Recently, ribosome biogenesis has emerged as an effective target in cancer therapy. A series of compounds that inhibit ribosome biosynthesis or function have shown their toxic action on cancer chemotherapy 35-37. Recent literatures of HCC ribosome profiling also provided insightful data resource for dissecting the translatome shift in liver cancer, at sub-codon resolution, and the regulatory mechanisms of oncogenic signaling and HCC therapy 38-40. In our study, we demonstrated that ribosome was the vital signaling pathway in accordance to PIGR expression changes. Ribosome genes changed in line with PIGR *in vitro*. Unfortunately, we did not perform experiments *in vivo* and potential connections between PIGR and ribosome protein still needs to be investigated.

Considered the critical roles of PIGR and ribosome in the development of human malignancies, we cautiously assumed that PIGR might exert oncogenic functions through simulating activation of ribosome pathway in the progression of HCC.

## Figures and Tables

**Table 1:** Primer sequence of genes screened by transcriptome sequencing analysis

Gene	Forward	Reverse
RPL10	GATGCCAAGATTCGCATTTTTG	AGCTGCTCATATTCATCTGACA
RPL10A	GATCAAGCAGATTCCACGAATC	GAACTTGATTGTGGACTTCACC
RPL12	ACCACCAAGAGACAGAAAGAAA	CGAGCAATGTTGACAATCTCAT
RPL19	TCACATGGATGAGGAGAATGAG	CGCTTGTTTTTGAACACATTCC
RPL36	CAAAGTGACCAAGAACGTGAG	TCCCCACCCTTTTCTTGATAAA
RPL38	GGATGCCAAATCTGTCAAGATC	TTCTCTTTGTCAGTGATGACCA
RPL41	GCCGTAGACGGAACTTCGCC	TCTGCTCCTGTGGCCTCCAC
RPL6	CATGTATTCCAGAAAGGCCATG	GCATTTTGCGAAGTTTAACCAC
RPL8	TTTGTGTATTGCGGCAAGAAG	GAGATAACGGTGGCATAGTTCC
RPS12	CCAACTGTGATGAGCCTATGTA	TTTACAAAGGCCTACCCATTCT
RPS14	GAGAATGTATTTGGTGTCTGCC	GTTTCCTTGCCAGAAAGATCAG
RPS15A	CTCACTGTGATGATGAAGCATG	AGTACAATGAAACCAAACTGGC
RPS2	GCCAAGCTCTCCATCGTC	GTGCAGGGATGAGGCGTA
RPS27A	CTGGAAGATGGACGTACTTTGTC	CGACGAAGGCGACTAATTTTGC
RPSA	GTGGCACCAATCTTGACTTCC	GCAGGGTTTTCAATGGCAACAA
